# Unlocking public health competencies: the dose–response effect of problem-based learning on undergraduate student outcomes

**DOI:** 10.3389/fpubh.2025.1560098

**Published:** 2025-05-08

**Authors:** Ashley Falcon, Andrew Porter, Yui Matsuda, Cynthia L. Foronda, Padideh Lovan, Beck Graefe

**Affiliations:** School of Nursing and Health Studies, University of Miami, Coral Gables, FL, United States

**Keywords:** problem-based learning, public health, education, active learning, dose response, competency development, undergraduate

## Abstract

**Introduction:**

Problem-based learning (PBL) is a student-centered pedagogical strategy that emphasizes active learning through the exploration of complex real-world problems. While it has demonstrated effectiveness in undergraduate- and graduate-level programs, there is a notable gap in research on the dose–response relationship between the extent of PBL exposure and competency development in public health education. This study examines the effect of PBL on undergraduate public health students’ learning outcomes and explores the dose–response relationship between the extent of PBL exposure and the development of specific public health competencies in students.

**Methods:**

A pre-posttest design using surveys evaluated the impact of PBL across three undergraduate public health courses at a medium-sized, private university in South Florida. Students (*n* = 150) completed surveys at the beginning and end of each course to assess changes in 10 skill domains. Composite scores were calculated for each domain and overall competency. Repeated samples t-tests assessed pre-to-post course improvements, and linear regression analyses examined the dose–response effect of the number of PBL courses taken on competency development. Post-hoc analyses examined gender and racial/ethnic differences.

**Results:**

Significant increases were found across all 10 learners’ skill domains from pre- to post-course measurements, with the most substantial gains in Data Literacy (Cohen’s *d* = 0.812), Critical Thinking (Cohen’s *d* = 0.726), and Public Speaking (Cohen’s *d* = 0.672). The number of PBL courses taken significantly predicted the total skill competency score (*β* = 0.24, *p* = 0.02), indicating a dose–response effect. Significant relationships were also found for individual domains such as Critical Thinking (*β* = 0.27, *p* = 0.009), Data Literacy (*β* = 0.24, *p* = 0.02), and Team Dynamics (*β* = 0.25, *p* = 0.02).

**Conclusion:**

Findings demonstrate that PBL effectively enhances several competencies critical to public health practice among undergraduate students. The novel discovery of a dose–response relationship suggests that increased exposure to PBL may lead to cumulative improvements in competencies. These results support the integration of multiple PBL courses in undergraduate public health curricula.

## 1 Introduction

Problem-based learning (PBL) is a transformative pedagogical strategy that emphasizes student-centered learning through the exploration of complex, real-world problems (1). Unlike traditional didactic instruction where students passively receive information, PBL actively engages students in self-directed learning through participation, collaboration, and reflection (2). Students are challenged to tackle complex real-world problems that lack clear, predefined solutions, thereby fostering critical thinking, problem-solving abilities, and the application of knowledge to practical situations (2–4).

Implementing PBL is particularly pertinent in public health education, given the interdisciplinary and applied nature of public health challenges that require a workforce equipped with both the soft and hard skills essential for fulfilling foundational competencies (5). In public health education, competency frameworks guide the development of essential skills and knowledge that prepare students for the workforce. In the United States, the Council on Education for Public Health (CEPH) outlines foundational competencies and cross-cutting concepts for standalone undergraduate public health education, including communicating effectively across diverse audiences and avenues of media, appropriate handling of public health information, critical thinking, personal worth ethic, teamwork, professionalism, and leadership (6). Globally, frameworks such as the World Health Organization’s Roadmap for Public Health and Emergency Workforce further underscore the need for these competencies, ensuring relevance beyond U.S. educational contexts (7).

Problem-based learning was originally developed in the 1960s and 1970s in medical education by Howard Barrows, who emphasized that even limited integration of PBL could yield substantial improvements in critical thinking and clinical reasoning skills (8, 9). Over the past several decades, a robust body of research has accumulated demonstrating the effectiveness of PBL in fostering student motivation, collaborative learning, and deeper understanding (10). Recent literature continues to underscore the effectiveness of PBL in enhancing soft skills (e.g., communication, teamwork, and leadership) and hard skills (e.g., data analysis, program evaluation) alike as it creates self-directed learning opportunities that promote overall proficiency for both undergraduate and graduate students (11–17). Despite these documented benefits of PBL, its adoption in public health education has been more recent and limited, and more research is needed to promote its utilization. In particular, there is a lack of research exploring dose–response - that is, how the quantity of PBL course exposures influences the extent of skill development and competency in students. Understanding whether increased exposure to PBL correlates with greater improvements in competencies can guide educators in structuring programs that maximize student learning outcomes.

The purpose of the study is to evaluate the impact of PBL with undergraduate public health students. The aims of the study are to: (1) examine the perceived impact of PBL on students’ learning outcomes across 10 skill domains, and (2) explore the dose–response relationship between the extent of PBL course exposure and the perceived development of specific public health competencies in students.

## 2 Materials and methods

### 2.1 Study design and participants

A pre-posttest design using surveys was employed to evaluate PBL across three core courses within an undergraduate public health degree program at a medium-sized, private university in South Florida. Students enrolled in these courses during the Spring 2024 semester were eligible to participate in the study. Participation in the study was voluntary, and all participants provided informed consent. Given that this study did not meet the definition of human research under 45 CFR 46, Institutional Review Board approval was not required.

### 2.2 PBL course descriptions

Within the undergraduate public health degree program, all courses with existing PBL integration were selected for this study. This included three required courses that span different levels of and topics within the curriculum: BPH206-Introduction to Public Health, BPH305-Issues in Health Disparities, and BPH309-Health and Environment. Integration of PBL varied by course. Three group-based PBL modules spanning 7.5 h of class were provided within the introductory public health course. PBL within the health disparities course consisted of five group-based PBL modules over the course of the semester with 9 h of dedicated in-class time. Students in the environmental health course participated in four group-based PBL modules over the course of the semester, totaling 8.75 h of in-class time. While the public health program is not structured as a formal cohort model, some students encounter one another across multiple courses due to self-selection and overlapping course enrollment patterns.

### 2.3 Instrument development

Three doctoral-prepared faculty with public health backgrounds and trained in PBL as part of a faculty fellowship developed a brief survey instrument assessing PBL competencies. Faculty developers aligned the survey instrument with CEPH foundational competencies and applicable cross-cutting concepts for standalone undergraduate public health programs (6). The survey instrument, which consisted of 24 items categorized into 10 distinct skill domains linked to the expected outcomes of PBL, solicited self-report data regarding students’ perceptions of their own competency development. Assessed domains included Communication Proficiency, Communication Versatility, Critical Thinking, Public Speaking, Data Literacy, Growth and Adaptability, Reflection, Listening, Team Dynamics, and Leadership. Items were scored on a five-point Likert scale, with descriptors assessing either level of agreement, level of confidence, or frequency of performing target skills.

### 2.4 Data collection and procedure

Data was collected using the Qualtrics online survey platform (18). Course instructors administered the anonymous, self-report survey during class time at the beginning and end of each course by providing a survey link. Students were asked to self-report their perceived skill levels in each of the 10 skill domains. Additionally, demographic information, including age, gender, race/ethnicity, and academic background, was collected to provide context for the analysis. In addition to the survey, students’ course evaluation comments related to PBL were collected at the end of the semester as part of standard university procedures. While PBL was not formally analyzed in these evaluations, these qualitative responses were reviewed and summarized to provide contextual insight into student perceptions of PBL and complement survey data.

### 2.5 Statistical analyses

Statistical analyses were performed using IBM SPSS Statistics, Version 29 (19). While the survey utilized Likert-type items, responses were treated as continuous variables in the analysis. This approach is supported by methodological research indicating that parametric statistical techniques such as *t*-tests and linear regression can be appropriately applied to Likert-type data, particularly when composite scores are used and distributions approximate normality (20). Moreover, simulation studies have shown that violations of the ordinal nature of Likert data have minimal impact on Type I error and power when assumptions are reasonably met, and sample sizes are adequate (21, 22).

Composite scores for each skill domain were computed by calculating the mean of the items within each domain. A composite score for all items was computed by calculating the mean of all items. To assess the impact of PBL on students’ competencies, repeated samples *t*-tests were conducted to compare pre-and post-course measurements for each of the 10 skill domains. Cohen’s d was calculated to estimate effect sizes, with values categorized as small (*d* = 0.2), medium (*d* = 0.5), or large (*d* ≥ 0.8).

In addition to analyzing changes within individual domains, we explored dose–response effects by examining whether the number of PBL courses taken by a student predi cted overall skill competency. A total skill competency score was created by averaging the means of all 10 skill domains. Linear regression models were then performed to assess the relationship between the number of PBL courses and both the overall competency score and each individual domain score. Adjusted R^2^ values were reported to indicate the proportion of variance in competency scores explained by the number of PBL courses.

Post-hoc analyses included two-tailed independent samples *t*-tests to examine gender differences in post-test domain scores, excluding one participant who identified as non-binary. Racial/ethnic differences were assessed using one-way ANOVA across all domains, followed by Tukey post-hoc adjustments to determine significant group differences.

## 3 Results

### 3.1 Demographic characteristics

A total of 159 students were enrolled across the three PBL courses. Of these, 100% of students in BPH206 and BPH309 responded to the survey, while 83.9% responded in BPH305, which yielded an overall response rate of 94.3%. Within the sample, 93 students were enrolled in one PBL course, 33 were enrolled in two PBL courses, and 24 students were enrolled in all three PBL courses. Participants were predominantly female (79.3%), White (54.7%), and non-Hispanic (74.7%) with upper-class student status (58.7%). Sample characteristics are detailed in full in Table 1.

**Table 1:** Sample characteristics.

Characteristic	N	%
Gender		
Female	119	79.3
Male	29	19.3
Non-binary/third gender	2	1.3
Race		
American Indian/Alaska Native	2	1.3
Asian/Pacific Islander	18	12.0
Black/African American	30	20.0
White/Caucasian	82	54.7
Mixed race	10	6.7
Not listed	5	3.3
Prefer not to say	3	2.0
Ethnicity		
Hispanic	37	24.7
Non-Hispanic	112	74.7
Prefer not to say	1	0.7
Class status		
Freshman	24	16.0
Sophomore	38	25.3
Junior	51	34.0
Senior	37	24.7
Sample size by class		
Introduction to public health	52	34.7
Issues in health disparities	47	31.3
Health and environment	51	34.0

### 3.2 Aim 1: impact of PBL on learning outcomes

Results from the repeated samples t-tests supported the first alternative hypothesis, indicating a significant increase in all skill domains from pre- to post-measurements for PBL effectiveness (Table 2). The most substantial gains were observed in Data Literacy (Cohen’s *d* = 0.812, *p* < 0.001), Public Speaking (Cohen’s *d* = 0.672, *p* < 0.001), and Critical Thinking (Cohen’s *d* = 0.726, *p* < 0.001) with medium to large effect sizes. Communication Proficiency (Cohen’s *d* = 0.662, *p* < 0.001) and Communication Versatility (Cohen’s *d* = 0.634, *p* < 0.001) exhibited significant increases with medium effect sizes, while Growth and Adaptability (Cohen’s *d* = 0.362, *p* = 0.001) and Listening (Cohen’s *d* = 0.357, *p* = 0.001) showed improvement with smaller effect sizes. Team Dynamics (Cohen’s *d* = 0.309, *p* = 0.004) and Leadership (Cohen’s *d* = 0.284, *p* = 0.008) demonstrated modest gains with smaller effect sizes. Qualitative feedback from students’ course evaluations included comments emphasizing the positive impact of PBL on critical thinking, teamwork, public speaking, and communication skills. Comments highlighted that PBL fostered more active learning, promoted problem-solving, and made class discussions engaging and relevant. Several students reported improvements in group collaboration, learning how to advocate for their beliefs during group discussions, and enhanced comfort navigating ambiguity.

**Table 2:** Repeated samples *t*-test results on all skill domains.

					95% CI	
PBL domain	Mean difference	*t*	*p*	Cohen’s *d*	Lower	Upper
Communication proficiency	0.496	6.346	**<0.001**	0.662	0.341	0.652
Communication versatility	0.489	6.085	**<0.001**	0.634	0.329	0.649
Critical thinking	0.549	6.963	**<0.001**	0.726	0.392	0.706
Public speaking	0.567	6.446	**<0.001**	0.672	0.392	0.742
Data literacy	0.582	7.791	**<0.001**	0.812	0.433	0.730
Growth and adaptability	0.232	3.475	**0.001**	0.362	0.099	0.364
Reflection	0.457	5.426	**<0.001**	0.566	0.289	0.624
Listening	0.239	3.428	**0.001**	0.357	0.101	0.378
Team dynamics	0.217	2.960	**0.004**	0.309	0.072	0.363
Leadership	0.239	2.719	**0.008**	0.284	0.064	0.414

Statistically significant findings are bolded.

### 3.3 Aim 2: dose–response relationship between PBL exposure and competency development

Linear regression results demonstrated that the second alternative hypothesis regarding a PBL dose–response effect was supported. Specifically, results indicated that the number of PBL courses taken significantly predicted the total skill competency score (*b* = 0.123, *t*(92) = 2.38, *p* = 0.02, 95% CI [0.02, 0.23]). The adjusted R^2^ value was 0.05.

Results of additional linear regression models fitted to examine the effect of the number of PBL courses on each skill domain individually are summarized in Table 3. For overall competency, the regression model indicated a significant positive relationship, with the number of PBL courses predicting an increase (*β* = 0.24, *p* = 0.02). Critical Thinking demonstrated a notable effect among the individual skill domains, with the number of PBL courses significantly predicting improvements in this area (*β* = 0.27, *p* = 0.009). Similarly, increases in Data Literacy (*β* = 0.24, *p* = 0.02) and Team Dynamics (*β* = 0.25, *p* = 0.02) were positively associated with the number of PBL courses. Listening (*β* = 0.23, *p* = 0.03) and Reflection (*β* = 0.21, *p* = 0.04) also showed significant improvements linked to PBL course participation.

**Table 3:** Linear regression test results for the number of PBL courses taken.

					95% CI		
Domain	*b*	SE	*β*	*p*	Lower	Upper	*r^2^*
Total competency	0.12	0.05	0.24	**0.02**	0.02	0.23	0.05
Communication proficiency	0.11	0.07	0.16	0.14	−0.03	0.25	0.01
Communication versatility	0.13	0.07	0.18	0.08	−0.02	0.27	0.02
Critical thinking	0.17	0.06	0.27	**0.009**	0.04	0.30	0.06
Public speaking	0.14	0.08	0.17	0.10	−0.03	0.30	0.02
Data literacy	0.15	0.06	0.24	**0.02**	0.03	0.28	0.05
Growth and adaptability	0.06	0.06	0.11	0.28	−0.05	0.18	0.002
Reflection	0.14	0.07	0.21	**0.04**	0.01	0.28	0.04
Listening	0.12	0.05	0.23	**0.03**	0.02	0.22	0.04
Team dynamics	0.15	0.06	0.25	**0.02**	0.03	0.28	0.05
Leadership	0.10	0.08	0.13	0.22	−0.06	0.26	0.006

Statistically significant findings are bolded.

Several domains - Communication Proficiency (*β* = 0.16, *p* = 0.14), Communication Versatility (*β* = 0.18, *p* = 0.08), Public Speaking (*β* = 0.17, *p* = 0.10), and Leadership (*β* = 0.13, *p* = 0.22) - exhibited positive but non-significant trends, with smaller effect sizes and less variance explained. Growth and Adaptability showed the weakest dose–response relationship (*β* = 0.11, *p* = 0.28).

Student feedback via course evaluations did not explicitly address the presence of a dose–response. Students did, however, describe positive impacts of repeated engagement in PBL activities. Specifically, students reported that recurring exposure to PBL fostered growing comfort with complex tasks, improved their performance on final presentations, and deepened team-based skills.

### 3.4 Post-test demographic comparisons

Results of the independent samples t-test revealed higher means for students who identified as women for the Listening domain [*t*(17.37) = 2.09, *p* = 0.05, Cohen’s *d* = 0.71] and the Team Dynamics domain [*t*(90) = 2.13, *p* = 0.04, Cohen’s *d* = 0.60]. No other comparisons were significant for gender. Similar tests looking at ethnicity revealed no differences for any domain or for the overall effect.

One-way ANOVA results indicated differences based on race for the Reflection domain only [*F*(5, 87) = 3.01, *p* = 0.02, $\eta^{2}=0.15$ ], with a large magnitude of effect (Table 4). Post-hoc analysis using Tukey adjustment showed that all racial groups except for “Prefer not to say” had significantly higher means compared with “Mixed Race” (Asian/PI: MD = 0.96, *p* = 0.022; Black/AA: MD = 0.92, *p* = 0.045; White/Caucasian: MD = 0.85, *p* = 0.023; Not Listed: MD = 1.22, *p* = 0.018). No significant differences were found based on class status or major. *Post hoc* results for significant overall effects are summarized in Table 5.

**Table 4:** Results of ANOVA tests for post-test PBL skill domains based on demographic factors.

	Race			Class Status			Major		
Measure	F	*p*	*η* ^2^	*F*	*p*	*η* ^2^	*F*	*p*	*η* ^2^
Total competency	1.71	0.14	0.09	1.24	0.3	0.04	0.32	0.72	0.01
Communication proficiency	1.94	0.1	0.1	0.72	0.54	0.02	0.04	0.96	0
Communication versatility	2	0.09	0.1	1.86	0.14	0.06	0.16	0.86	0.01
Critical thinking	0.34	0.89	0.02	0.65	0.59	0.02	0.15	0.87	0
Public speaking	1.7	0.14	0.09	0.24	0.87	0.01	0.01	0.99	0
Data literacy	1.87	0.11	0.1	0.75	0.53	0.03	0.47	0.63	0.01
Growth and adaptability	1.03	0.4	0.06	1.46	0.23	0.05	0.16	0.85	0.01
Reflection	3.01	**0.02**	0.15	2.12	0.1	0.07	0.46	0.63	0.01
Listening	1.41	0.23	0.08	0.68	0.57	0.02	1.41	0.25	0.04
Team dynamics	1.11	0.36	0.06	1.13	0.34	0.04	2.08	0.13	0.06
Leadership	0.74	0.59	0.04	0.19	0.91	0.01	1.68	0.19	0.05

Statistically significant findings are bolded.

**Table 5:** Post-hoc comparisons* for the Reflection domain based on race.

			95% CI	
Pairwise comparison	Mean difference	*p*	Lower	Upper
API/Black or African American	0.04	1.00	−0.68	0.76
API/White or Caucasian	0.11	0.99	−0.42	0.65
API/Mixed race	0.96	0.02	0.09	1.83
API/No race indicated	−0.26	0.96	−1.19	0.67
API/Prefer not to say	−0.26	0.99	−1.4	0.88
Black or African American/White or Caucasian	0.07	0.99	−0.52	0.66
Black or African American/Mixed race	0.92	0.05	0.01	1.83
Black or African American/No race indicated	−0.3	0.94	−1.27	0.66
Black or African American/Prefer not to say	−0.3	0.97	−1.47	0.86
White or Caucasian/Mixed race	0.85	0.02	0.08	1.62
White or Caucasian/No race indicated	−0.38	0.78	−1.21	0.46
White or Caucasian/Prefer not to say	−0.38	0.91	−1.44	0.69
Mixed race/No race indicated	−1.22	0.02	−2.3	−0.14
Mixed race/Prefer not to say	−1.22	0.06	−2.49	0.04
No race indicated/Prefer not to say	0	1.00	−1.31	1.31

*Tukey Adjustments were used.

## 4 Discussion

Study results suggest that PBL is effective in enhancing a range of skills that are prerequisites for public health tasks. Our findings are consistent with and build on the broader literature indicating that PBL facilitates significant improvements in various skill domains, such as critical thinking, problem-solving, data analysis, and communication—all of which are essential for effective public health practice (14, 23). The significant improvements observed across all measured domains from pre- to post-course evaluations enhance our understanding of how PBL can effectively advance student learning outcomes. The competencies evaluated in this study, while self-reported and personal in nature, align closely with the CEPH competencies and directly support task-based public health competencies (6). Our findings suggest that PBL has the potential to cultivate these competencies, supporting not only academic development but also workforce preparedness. Future research may benefit from more explicitly measuring task-based performance outcomes alongside perceived competency gains to fully capture the impact of PBL.A noteworthy aspect of our results is the presence of a dose–response effect, wherein the number of PBL courses taken correlates positively with the level of competency achieved. This novel finding suggests that repeated exposure to PBL may enhance learning outcomes in a cumulative fashion, emphasizing the potential benefit of integrating PBL throughout the public health curriculum. The adjusted R^2^ values observed in this study provide further insight into the dose–response relationship. For example, the adjusted R^2^ of 0.05 for the total skill competency model suggests that 5% of the variance in perceived competencies can be attributed to the number of PBL courses a student completed. Although this may appear modest, in educational and behavioral sciences, where numerous factors influence outcomes, even small effect sizes and R^2^ values can be practically significant. Moreover, the consistency of significant predictive relationships across several domains (e.g., Critical Thinking, Data Literacy, and Team Dynamics) highlights the relevance of repeated PBL exposure in fostering these specific competencies. Nonetheless, curricular integration efforts intended to maximize the benefits of PBL should factor in other relevant considerations, including faculty and student preferences for other modalities or hybrid models that incorporate other effective pedagogical strategies (24).

Subpopulation analyses suggest that the overall uptake of skills across domains was largely equitable, yielding learning gains for all students. Results based on gender and race imply some variability in these gains. For instance, higher competency scores in domains such as Listening and Team Dynamics among female students, as well as differences based on race for the Reflection domain, indicate the potential for differential impacts of PBL based on these demographic characteristics. While our study found no ethnicity-specific differences, a previous study of PBL among immigrant students noted different learning style preferences based on ethnicity, though PBL was still shown to be an effective pedagogy for all (25). Other personal factors, such as the level of student motivation, may influence the degree of benefit derived from PBL (26). Further research is needed to explore these potential differences and determine how best to tailor PBL approaches accordingly to maximize benefits across diverse student groups.

### 4.1 Limitations

Results should be considered within the context of the study’s limitations. This study utilized self-reported responses of perceived, rather than objectively measured, competency changes. Further, the study did not include a formal control group of students who were not exposed to PBL. As such, caution should be exercised when attributing these changes solely to the PBL approach, as other external factors could have influenced the observed results. Future studies with a control group and multiple forms of assessment, both objective and subjective, would provide a more robust framework for assessing the impact of PBL and whether gains translate into improved task-based performance. Generalizability of the findings may be limited due to the single-site, private institution setting, as well as by the predominantly female, Caucasian, and non-Hispanic composition of the sample.

The statistical power to detect significant differences in both the overall analyses (*t*-tests and ANOVAs) and subpopulation comparisons was limited. Specifically, *post hoc* power analyses demonstrate enough statistical power to detect only medium and large effects in t-tests and large effects in ANOVAs. Though many of the effect sizes for PBL skill domains were medium and large, this limitation suggests that smaller or more subtle differences, particularly for subpopulation comparisons, may have gone undetected. As such, caution is needed when interpreting these findings, especially in relation to potential differences that were not statistically significant. In light of this limitation, subpopulation comparisons should be considered exploratory, and further research with larger, more diverse samples is necessary to determine whether these differences are consistent across broader populations.

Nonetheless, this study’s strengths include its longitudinal design and the systematic assessment of the dose–response relationship, which contribute valuable insights into the pedagogical effectiveness of PBL. Further exploration of the variability in PBL formats and the longitudinal impact of PBL on student competencies will be critical in understanding and maximizing the benefits of the pedagogy. Additionally, the variability in PBL implementation across courses could have influenced the outcomes, and the reliance on self-reported measures introduces the potential for response bias. This underscores the importance of developing and utilizing validated measurement instruments in future research to ensure accuracy and reliability in assessing competency development.

### 4.2 Impact and recommendations

The impact of PBL on developing public health competencies is profound and extends beyond mere knowledge acquisition to include enhanced engagement and applicability in practical settings. This study underscores PBL’s value in creating more dynamic and contextually relevant learning environments that better prepare students for the complexities of public health careers. PBL offers an engaging and practical approach to education that prepares students for real-world public health challenges by fostering both soft and hard skills that are the foundation of public health competencies necessary for effective practice in the field. The demonstration of a dose–response effect further emphasizes the value of integrating PBL throughout the curriculum.

Given the positive outcomes associated with PBL documented in this study, several recommendations can be made for educators and curriculum developers in public health. First, the integration of PBL should be considered not just as a supplementary educational strategy but as a core component of public health education. Additionally, future research should aim to validate the scales used for measuring competencies in PBL settings, compare PBL with traditional teaching methods, and explore different PBL formats to determine the most effective approaches for various learning contexts.

While the findings of this study highlight the potential benefits of PBL in fostering essential public health competencies, it is important to emphasize that the success of PBL, as with any innovative pedagogical approach, requires significant commitment in terms of training, faculty skill development, and appropriate resources. Instructors must be well-trained in facilitating PBL effectively, which requires time and expertise beyond traditional teaching methods (27–29). Additionally, PBL relies on the availability of resources such as structured learning environments, adequate support for collaborative group work, and access to real-world data and case studies (28). Without these key elements, the effectiveness of PBL may be diminished, and students may not fully realize the intended learning outcomes.

The shift toward more resource-intensive pedagogies like PBL may be perceived as a challenge, particularly in light of the economic advantages of traditional, large-scale lecture formats. However, the long-term benefits of PBL, including the development of critical thinking, teamwork, and problem-solving skills, position it as a valuable investment for public health education. Public health educators, administrators, and policymakers must consider these factors when advocating for the integration of PBL into curricula. To ensure the sustained success of PBL, further research should explore the cost-effectiveness of PBL implementation, as well as strategies to balance resource allocation with the potential benefits of improved educational outcomes (28, 29).

## 5 Conclusion

This study illuminates the potential benefits of PBL in undergraduate public health education. By highlighting perceived improvements across critical competencies, our findings position PBL as a promising pedagogical approach for fostering student engagement and enhancing professional preparedness. The novel discovery of a dose–response relationship suggests that increased exposure to PBL may lead to cumulative improvements in competencies. The positive outcomes observed across diverse skill domains underscore PBL’s potential to revolutionize educational experiences, making learning more dynamic, relevant, and impactful. Educators, curriculum developers, and academic institutions should embrace and integrate PBL more extensively within public health programs. By doing so, we can equip future public health professionals with the essential skills, confidence, and adaptability needed to tackle complex health challenges. PBL integration is an opportunity to innovate education, inspire our students, and advance the field of public health through transformative teaching and learning practices.

## Data Availability

The raw data supporting the conclusions of this article will be made available by the authors, without undue reservation.
